# Role of Age-Related Changes in DNA Methylation in the Disproportionate Susceptibility and Worse Outcomes of Sepsis in Older Adults

**DOI:** 10.3389/fmed.2022.822847

**Published:** 2022-02-15

**Authors:** Xiabing Lang, Lingling Shen, Tingting Zhu, Wenjun Zhao, Yang Chen, Chaohong Zhu, Qun Su, Cuili Wang, Yucheng Wang, Francesco Neri, Hong Jiang, Jianghua Chen

**Affiliations:** ^1^Kidney Disease Center, The First Affiliated Hospital, College of Medicine, Zhejiang University, Hangzhou, China; ^2^Key Laboratory of Nephropathy, Hangzhou, China; ^3^Institute of Nephropathy, Zhejiang University, Hangzhou, China; ^4^Zhejiang Clinical Research Center of Kidney and Urinary System Disease, Hangzhou, China; ^5^Critical Care Medicine Department, The First Affiliated Hospital, College of Medicine, Zhejiang University, Hangzhou, China; ^6^Life Sciences and Systems Biology Department, University of Turin, Turin, Italy

**Keywords:** sepsis, DNA methylation, aging, susceptibility, outcomes

## Abstract

Sepsis, a complex multisystem disorder, is among the top causes of hospitalization and mortality in older adults. However, the mechanisms underlying the disproportionate susceptibility to sepsis and worse outcomes in the elderly are not well understood. Recently, changes in DNA methylation have been shown to be linked to aging processes and age-related diseases. Thus, we postulated that age-related changes in DNA methylation may play a role in the onset and prognosis of sepsis in elderly patients. Here, we performed genome-wide methylation profiling of peripheral blood from patients with sepsis and controls. Among the CpG sites whose methylation changes may contribute to an increase in sepsis susceptibility or mortality, 241 sites that possessed age-related changes in DNA methylation in controls may partly explain the increased risk of sepsis in older adults, and 161 sites whose methylation significantly correlated with age in sepsis group may be the potential mechanisms underlying the worse outcomes of elderly septic patients. Finally, an independent cohort was used to validate our findings. Together, our study demonstrates that age-related changes in DNA methylation may explain in part the disproportionate susceptibility and worse outcomes of sepsis in older adults.

## Introduction

Sepsis, a life-threatening organ dysfunction characterized by a dysregulated host response to infection, is a leading cause of morbidity and mortality among critically ill patients (1). The incidence of sepsis is expected to increase, likely due to the aging of our population (2, 3). More than 60% of sepsis occur in patients aged ≥65 years, and about 60% of in-hospital mortality from sepsis occur in this age group (4, 5). Aging has been recognized as the primary risk factor for developing sepsis (6). However, the underlying mechanisms of the disproportionate susceptibility to sepsis and worse outcomes in older adults are not yet fully understood.

Epigenetic alterations, especially changes in DNA methylation, have been found to be associated with a variety of diseases, including sepsis (7–9). Several studies have revealed differential methylation profiles which were correlated with sepsis status both in adults and neonates (10–12). Recently, we found that inhibiting DNA methylation by Decitabine can mitigate the effects of sepsis and improve survival of mice by attenuating the activation of NF-κB signaling pathway and down-regulating inflammatory cytokine levels. (13). In addition, changes in DNA methylation also have emerged as a biomarker of aging (14). Approximately 2–14% of the CpG sites show age-associated changes, either hypermethylation or hypomethylation with increasing age (15). Interestingly, age-related changes in DNA methylation have been linked to various diseases, such as diabetes (16), kidney diseases (17), and cancer (18–20). Nevertheless, age-related DNA methylation changes have not been studied in the context of sepsis.

In this study, we analyzed genome-wide methylation profiling in peripheral blood from sepsis patients and controls to explore sepsis and outcome-related CpG sites. Furthermore, we investigated DNA methylation patterns associated with subject age, and found some age-related DNA methylation changes which may explain in part the increase in sepsis incidence and mortality with age.

## Materials and Methods

### Patients and Samples Collection

All the patients included were from the First Affiliated Hospital, College of Medicine, Zhejiang University. Patients diagnosed of sepsis during the intensive care unit (ICU) period were enrolled in the study. Sepsis was defined according to the Third International Consensus Definitions for Sepsis and Septic Shock (Sepsis-3) (21). Detailed information of the selected patients was further inspected by a clinician. Controls were age-matched healthy volunteers. The hospital's Institutional Review Committee on Human Research approved the study protocol and all of the patients provided written informed consent.

Follow-up blood samples were obtained within the first 24h after admission. Peripheral blood was collected into EDTA-containing tubes. DNA was extracted using QIAamp® DNA Mini and Blood Mini kits (AXYGEN).

### DNA Preparation and Genome-Wide Methylation Profiling

The extracted DNA was bisulfite converted by Epitect Bisulfite kit (Qiagen, Germany) according to the manufacturer's instruction, 1–2 ug of genomic DNA was incubated with the bisulfite reactions in a thermocycler following the procedure of recommended bisulfite conversion thermal cycler conditions. The converted DNA could be combined to the column, washed, incubated with a desulfonation buffer, washed again and finally eluted with 20 μl of elution buffer. The quality and purity of the converted DNA was tested by Nanodrop.

Genome-wide methylation profiling was performed using Infinium HumanMethylation 450 BeadChip array (Illumina, San Diego, CA, USA). After whole-genome amplification with 200 ng of input bisulfite-converted DNA, the product was fragmented, purified and applied to the BeadChips using Illumina-supplied reagents and conditions. After extension, the array was stained fluorescently, and scanned with an iSan System (Illumina). Data were analyzed by GenomeStudio Methylation Module V1.8 Software (Illumina). A CpG site was considered to be informative if the sum of the signals for methylated and unmethylated sequence at the CpG site was significantly higher (detection *p*-value < 0.01) than signals of the negative control probes on the same array. For each CpG site, the β value reflects the methylation level, which was computed by β = (max (M, 0))/(|U| + |M|+100). A β value of 0–1.0 indicates the percent methylation from 0 to 100%, respectively.

### Pyrosequencing

The genomic DNA was bisulfite converted from unmethylated cytosine to uracil by using the Epitect Bisulfite kit (Qiagen). Primers were designed by the PyroMark Assay Design 2.0. PCR was performed with the following cycling conditions: initial denaturation step: 95°C for 3 min; 40 cycles of PCR in denaturation step: 94°C for 30 s; annealing step: Tm of primer; extension step: 72°C for 1 min and final extension: 72°C for 7 min. Subsequently, streptavidin coated sepharose beads, PyroMark Gold reagents (Qiagen), Pyrosequencing Vacuum Prep Tool and PyroMark Q96 software (Qiagen) were used for the determination and analysis of DNA methylation according to the manufacturers' instructions.

### Statistical Analyses

All statistical analyses were done using R (http://www.r-project.org/) and GraphPad Prism 8.0 (GraphPad Software, CA, USA). For methylation variable positions (MVPs) identification, we used the R limma package to establish a linear model and calculated the *p*-value. The Benjamini & Hochberg method was used to correct for multiple testing, and false discovery rate [FDR] < 0.05 was considered significant Sepsis outcome-related CpG sites were identified using the R limma package, and *p*-value < 0.05 was considered statistically significant. Functional enrichment analyses were performed by R package missMethyl (22) and Metascape (https://metascape.org/). The cutoff criteria was set at *p*-value < 0.05. We used default function lm() in R to construct the linear regression model, which was fitted by least-squares to DNA methylation of CpG sites and age. An association was considered significant where *p*-value < 0.05. Student's *t*-test was used for the comparison between groups in the validation cohort, and *p*-value < 0.05 was considered statistically significant.

## Results

### Sepsis-Associated Methylation Variable Positions

**Figure 1:**
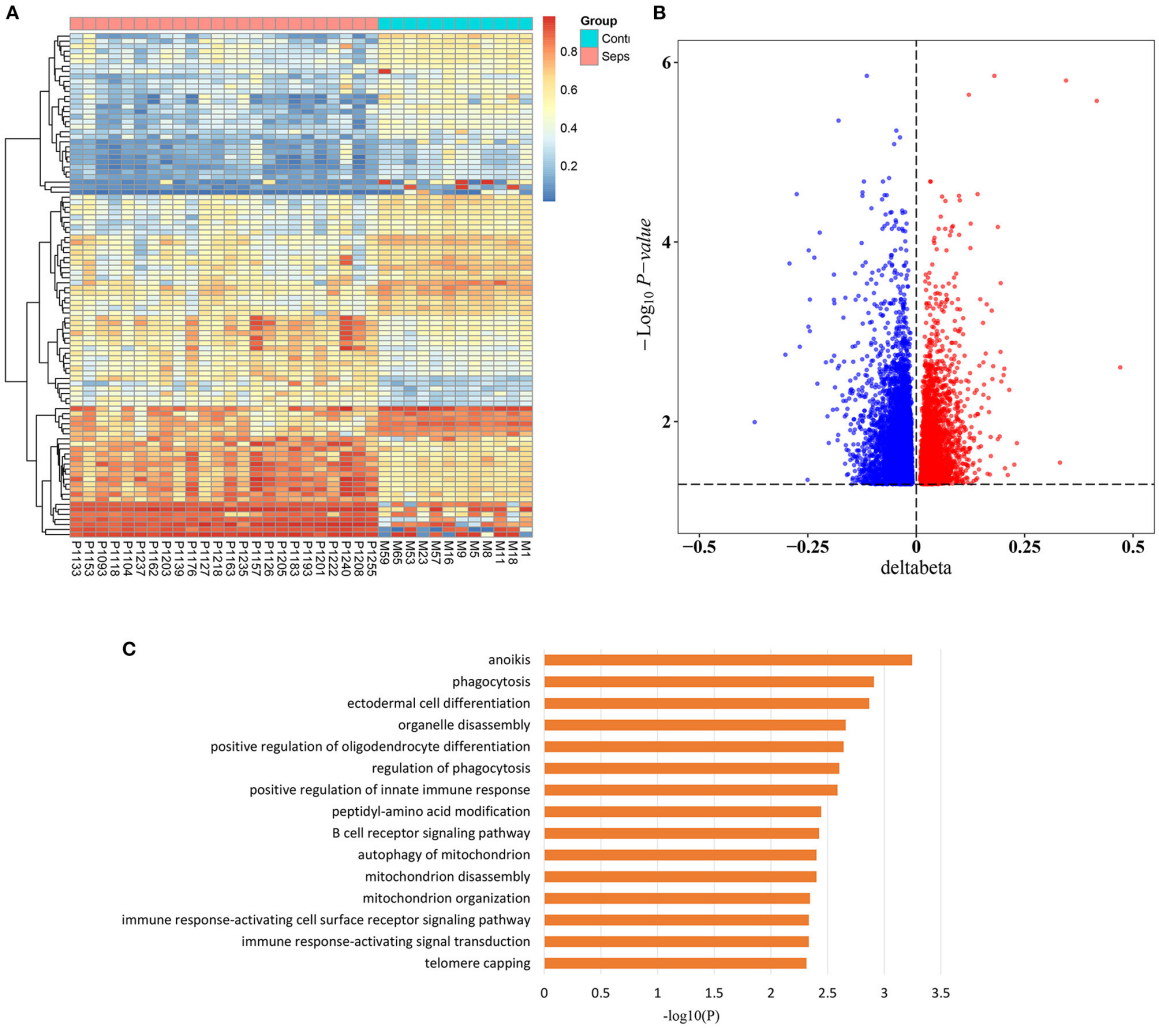
Sepsis-associated methylation variable positions. **(A)** Heatmap of top 100 methylation variable positions (MVPs) based on Δβ in sepsis patients and controls (FDR < 0.05). **(B)** Volcano plot of 8,437 MVPs. **(C)** The top 15 significant gene ontology (GO) biological process terms of sepsis-associated MVPs.

To investigate the epigenetic changes in sepsis, genome-wide methylation profiling was performed in peripheral blood from 24 sepsis patients aged 16–88 years, and 12 healthy controls aged 22–84 years (patient characteristics are shown in Supplementary Table 1). After quality control, batch correction, and normalization, a total of 8,437 CpG sites which significantly differentially methylated between sepsis and control group were identified as methylation variable positions (MVPs) (Supplementary Table 2). The top 100 MVPs are shown as an expression heatmap (Figure 1A). Principal component analysis (PCA) using the methylation data of all MVPs showed samples from sepsis patients and controls separated along the first principal component (Supplementary Figure 1). In all MVPs, 3,042 CpG sites (36.1%) were hypermethylated in sepsis patients, while 5,395 (63.9%) sites were hypomethylated (Figure 1B). However, only a small part of MVPs have relatively large Δβ (|Δβ|> 0.2), and focusing on those with large Δβ, we found that 6 of 26 differentially methylated genes (23.1%) were previously associated with sepsis in the literature (Supplementary Table 3), including ALK, ZEB2, MNDA, AIM2, TLR5, and JUNB. These genes are involved in various stages of sepsis, from immune response, to acute inflammation, to the regulation of apoptosis. More importantly, they have also been found to be implicated in aging process.

In addition, in order to unravel the potential biological function of all sepsis-associated MVPs, we performed Gene Ontology (GO) enrichment analysis (Figure 1C). The results demonstrated that they were mainly enriched in phagocytosis, immune response, and mitochondria, which are highly associated with sepsis (23–25).

### Role of Age-Related CpG Sites in the Disproportionate Susceptibility to Sepsis in the Elderly

**Figure 2:**
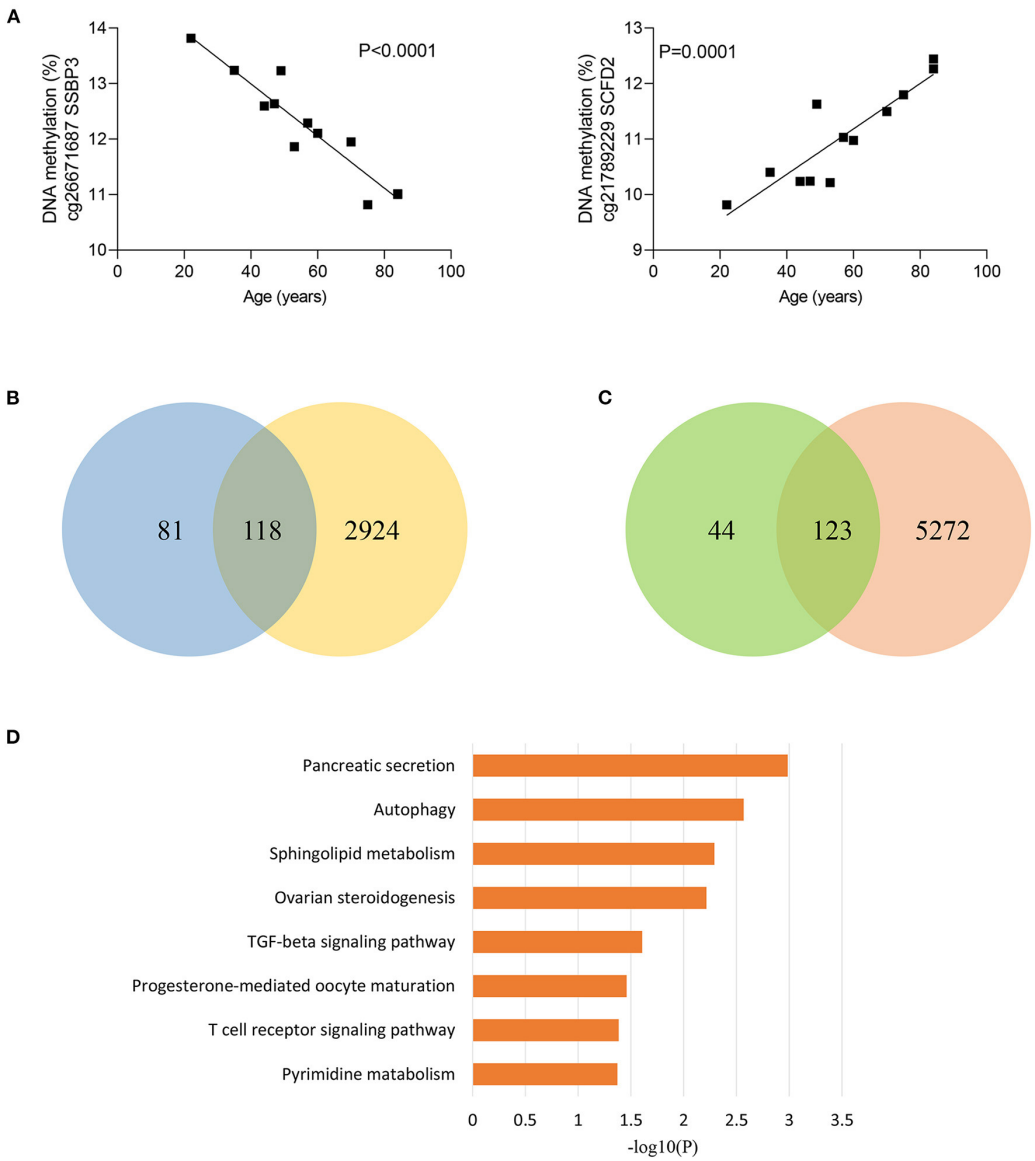
Age-related methylation changes in controls. **(A)** Correlation between age and methylation of two CpG sites with the most significant correlations with age in controls as analyzed by linear regression analysis (*n* = 12). **(B)** Venn diagram showing the intersection of the sets of hypermethylated sites with increasing age in controls and hypermethylated sites in sepsis. **(C)** Venn diagram showing the intersection of the sets of hypomethylated sites with increasing age in controls and hypomethylated sites in sepsis. **(D)** Kyoto Encyclopedia of Genes and Genomes (KEGG) pathway enrichment analysis of 227 genes distributed by 241 age-related CpG sites.

Considering that sepsis disproportionally affects the elderly, we next divided sepsis patients and controls into two age groups, younger than 50 years and older than 50 years. Of note, 5,87 of 84,37 CpG sites (69.8%) were differentially methylated between sepsis patients and controls under 50 years, while 8,012 of 8,437 CpG sites (95.0%) were differentially methylated between sepsis patients and controls over 50 years, supporting that aging is the primary risk factor for developing sepsis. Then, we tested to determine whether methylation of sepsis-associated MVPs are correlated with age. Then, we performed linear regression analysis and found that methylation of 366 sepsis-associated CpG sites were significantly associated with age in control group (Supplementary Table 4). The most significant correlations between DNA methylation and age are presented in Figure 2A. Next, we wondered whether sepsis- associated CpG sites were enriched in age-related CpG sites and performed Fisher's exact test. However, there was no statistical significance (*p* = 0.097, Supplementary Table 5). Of the 366 age-related CpG sites, 199 sites (54.4%) were hypermethylated with increasing age, and 167 sites (45.6%) were hypomethylated (Supplementary Figure 2). Moreover, we observed that 118 of 199 sites (59.3%) hypermethylated with age in control group exhibited an increased DNA methylation in sepsis patients (Figure 2B). Similarly, 123 of 167 sites (73.7%) hypomethylated with age in control group exhibited a decreased DNA methylation in sepsis patients (Figure 2C). Together, these findings indicated that 241 age-related CpG sites may play a mechanistic role in the disproportionate susceptibility to sepsis in older adults. Furthermore, we verified whether genes distributed by 241 CpG sites were enriched in specific pathways, and found that the TGF-β signaling pathway was the highest-ranked pathway (Figure 2D), which is involved in the pathogenesis of sepsis (26).

### Sepsis Outcome-Related CpG Sites

**Figure 3:**
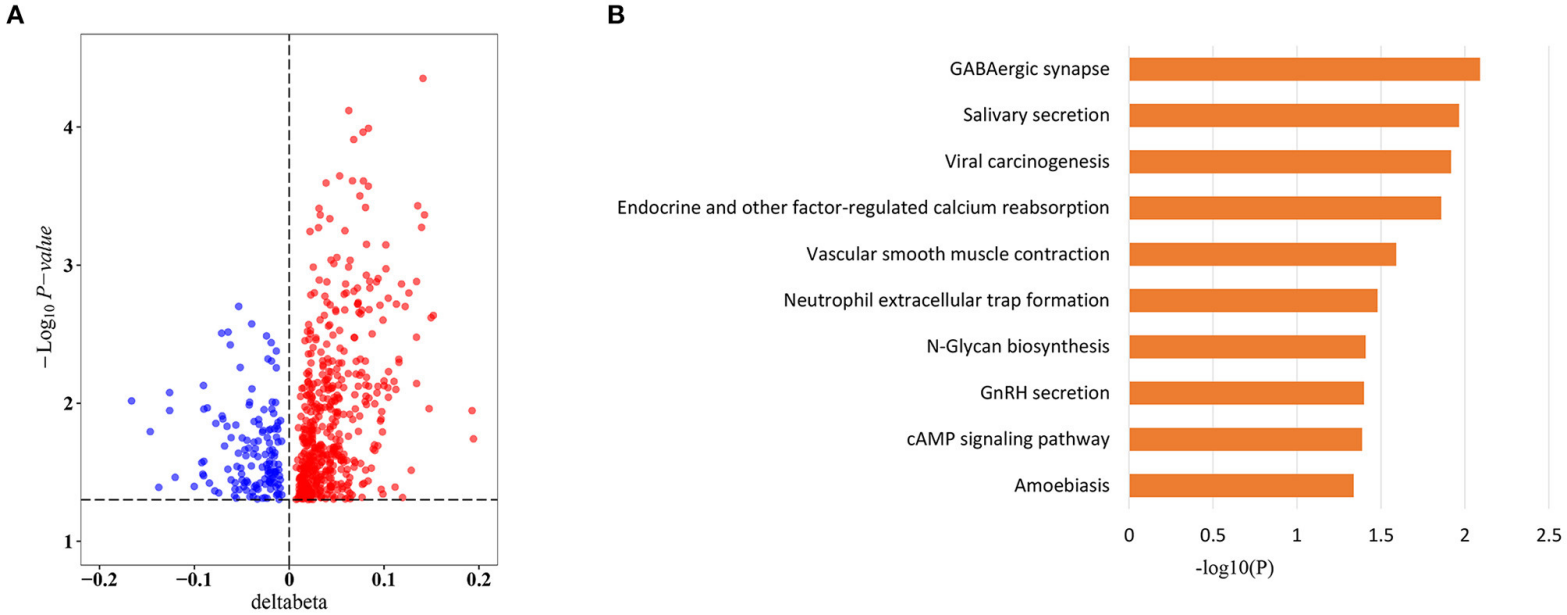
Sepsis outcome-related CpG sites. **(A)** Volcano plot of sepsis outcome-related CpG sites (*n* = 657). **(B)** Kyoto Encyclopedia of Genes and Genomes (KEGG) pathway enrichment analysis of sepsis outcome-related CpG sites.

To explore whether sepsis-associated MVPs were associated with sepsis outcomes, we divided sepsis patients into two groups (7 survivors and 17 non-survivors) according to their hospital outcomes. Intriguingly, 657 of 8,437 CpG sites were significantly differentially methylated between two groups (Supplementary Table 6), and more CpG sites were hypermethylated in non-survivors, with 514 (78.2%) hypermethylated vs. 143 (21.8%) hypomethylated CpG sites (Figure 3A). Next, we investigated which pathways were affected by the methylation changes that were associated with sepsis outcomes. Interestingly, these CpG sites were involved in cAMP signaling pathway (Figure 3B), which plays an important role in sepsis-induced organ dysfunction (27, 28).

### Role of Age-Related CpG Sites in the Worse Outcomes of Elderly Septic Patients

**Figure 4:**
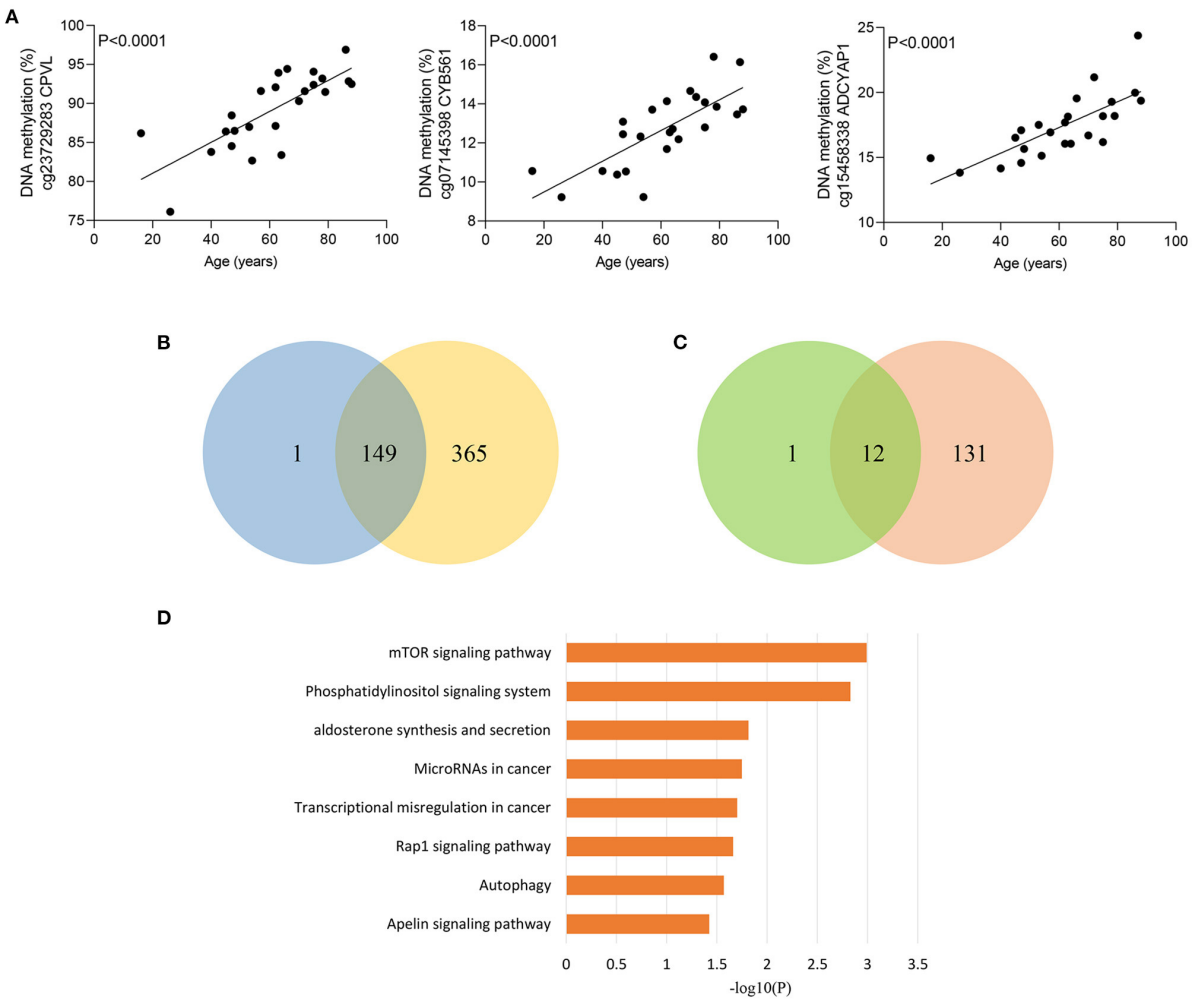
Age-related methylation changes in sepsis patients. **(A)** Correlation between age and methylation of three CpG sites with the most significant correlations with age in sepsis patients as analyzed by linear regression analysis (*n* = 24). **(B)** Venn diagram showing the intersection of the sets of hypermethylated sites with increasing age in sepsis and hypermethylated sites in non-survivors. **(C)** Venn diagram showing the intersection of the sets of hypomethylated sites with increasing age in sepsis and hypomethylated sites in non-survivors. **(D)** Kyoto Encyclopedia of Genes and Genomes (KEGG) pathway enrichment analysis of 155 genes distributed by 161 age-related CpG sites.

Given that elderly septic patients have increased hospital mortality than younger patients (29), we wondered whether methylation of sepsis outcome-related CpG sites were correlated with age in sepsis group. Strikingly, methylation of 163 CpG sites were significantly associated with age in sepsis patients (Supplementary Table 7). Figure 4A shows the most significant correlations between DNA methylation and age. Among the 163 CpG sites, more CpG sites were hypermethylated with age: 150 (92.0%) hypermethylated vs. 13 (8.0%) hypomethylated CpG sites. Moreover, we observed that 149 of 150 sites (99.3%) hypermethylated with increased age in sepsis group exhibited an increased DNA methylation in non-survivors (Figure 4B). A similar observation was made for 12 of 13 hypomethylated CpG site (92.3%) (Figure 4C). Together, these data suggested that 161 age-related CpG sites may play a crucial role in the worse outcomes of elderly septic patients. Furthermore, pathway analysis of the genes distributed by 161 CpG sites revealed that the mTOR signaling pathway was the top enriched pathways (Figure 4D), which is implicated in immunosuppression following sepsis and aging process (30, 31).

### Validation of the CpG Site in VAC14

**Figure 5:**
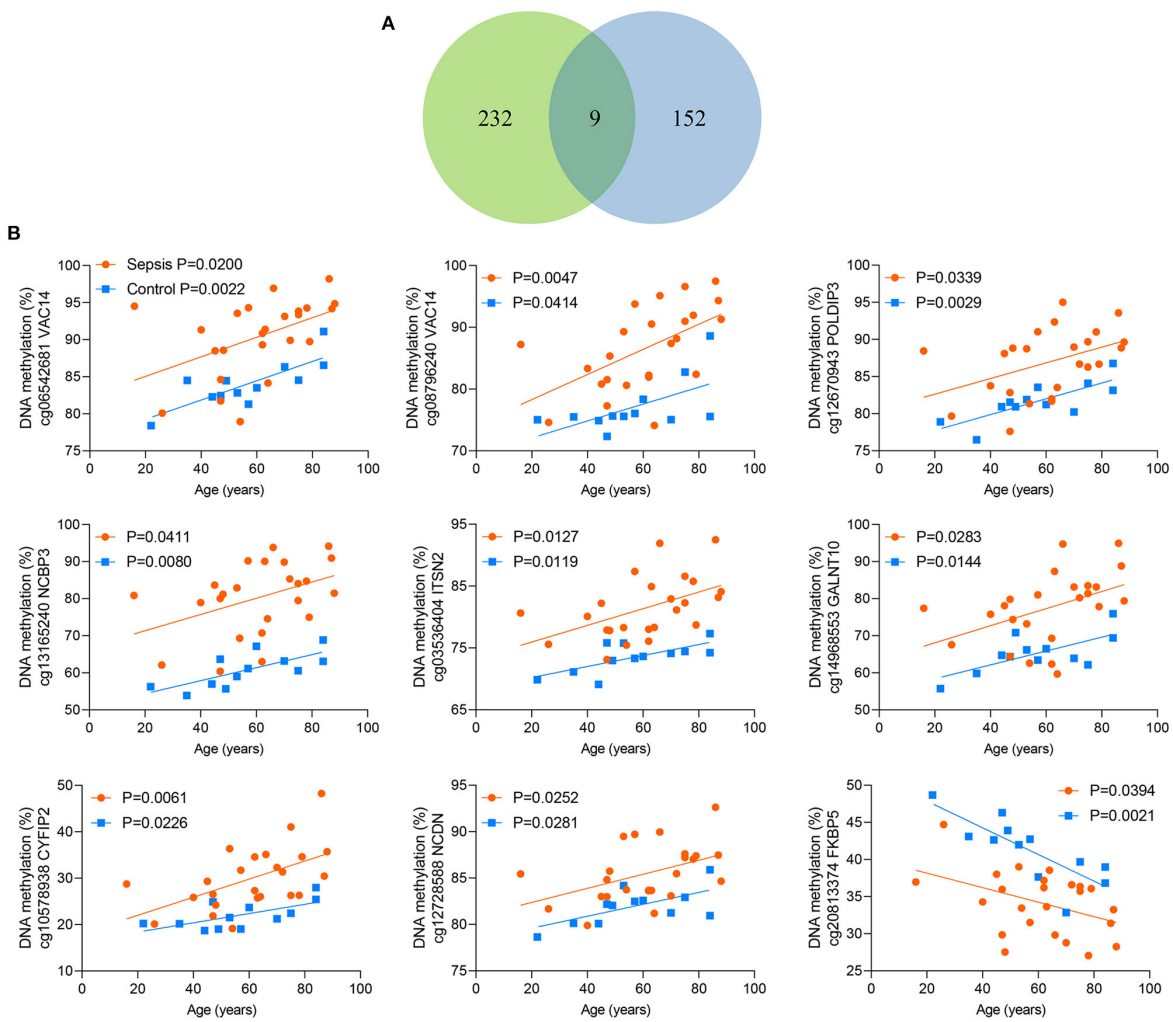
Nine shared CpG sites. **(A)** Venn diagram showing the intersection of the sets of 241 age-related CpG sites in controls and 161 age-related CpG sites in sepsis patients. **(B)** Correlation between age and methylation of 9 CpG sites as analyzed by linear regression analysis.

**Figure 6:**
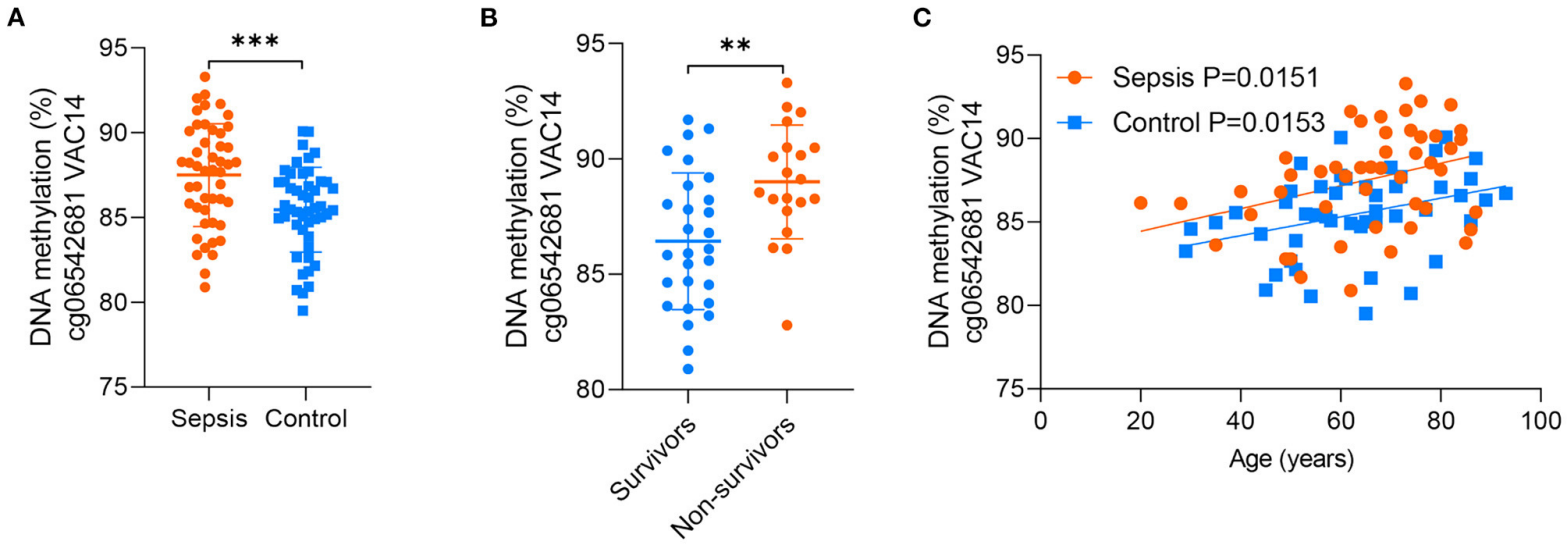
Validation of the CpG site in VAC14. **(A)** DNA methylation of the CpG site in VAC14 in peripheral samples from 48 sepsis patients and 48 controls were analyzed by the pyrosequencing assays. **(B)** 48 sepsis patients were divided into two groups (28 survivors and 20 non-survivors) according to their hospital outcomes. **(C)** Correlation between age and methylation of the CpG site in VAC14 as analyzed by linear regression analysis. Data shown are mean ± SD. **P < 0.01, ***P < 0.001. Comparisons between two groups were done by student's t-test.

Between the above two age-related CpG sets, 9 CpG sites were shared and directionally consistent (Figures 5A,B). Among the 9 sites, 2 CpG sites were distributed in VAC14, which is relevant to bacteremia secondary to multiple pathogens (32). Then, we selected one site (cg06542681) with the most significant association between DNA methylation and age in control group for further validation. The pyrosequencing assays were performed to analyze DNA methylation of the CpG site in VAC14 in peripheral blood taken from another cohort of 48 sepsis patients and 48 controls (patient characteristics are shown in Supplementary Table 8). As expected, this CpG site hypermethylated with increasing age and exhibited an increased DNA methylation both in sepsis patients and non-survivors (Figures 6A–C). Overall, our findings supported that age-related changes in DNA methylation may potentially contribute to the disproportionate susceptibility and worse outcomes of sepsis in the elderly.

## Discussion

**Figure 7:**
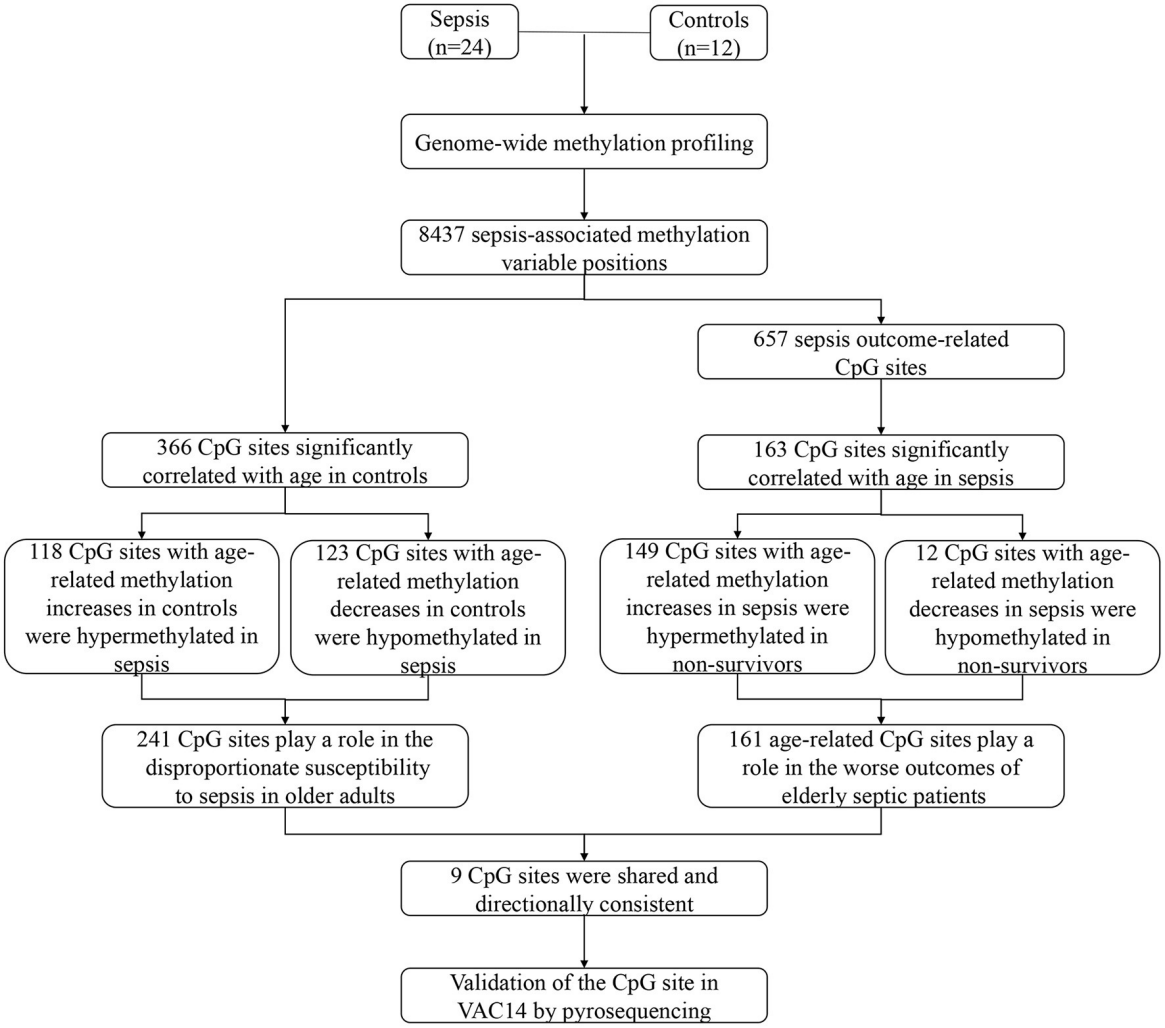
Schematic of study.

Our study shows the genome-wide methylation profiling of peripheral blood from adult patients with sepsis and healthy controls, and further provides the first sepsis-specific study of age-related DNA methylation changes. We identified some CpG sites whose methylation changes may contribute to an increase in sepsis susceptibility or mortality. Then, age-related DNA methylation changes wereexamined, which may provide new insights into the disproportionate susceptibility and worse outcomes of sepsis in the elderly. Indeed, 241 CpG sites whose methylation varies with age in controls may contribute to increased risk of sepsis in older adults. In addition, 161 CpG sites whose methylation changes significantly correlated with age in sepsis group may partly explain the poor prognosis of elderly septic patients (Figure 7). Together, our findings revealed for the first time that age-related changes in DNA methylation may play a mechanistic role in the increased susceptibility and mortality of sepsis in the elderly.

Epigenetic changes have been linked to key phases of sepsis, from the host-pathogen interaction to inflammatory response, to immune suppression, to organ failures (8). Our results demonstrated the existence of DNA methylation changes in sepsis patients vs. controls, supporting that epigenetic alterations may play an important role in the pathogenesis of sepsis. Furthermore, we revealed 657 CpG sites whose methylation changes were related to sepsis prognosis, with significantly more CpG sites hypermethylated in non-survivors. These findings are in line with our previous research that inhibiting DNA methylation may mitigate inflammation and improve survival in sepsis (13).

The current studies show that aging has a major impact on DNA methylation (33). Tens to hundreds of thousands of CpG sites show significant methylation changes (increase or decrease) with age. Several studies of anti-aging interventions demonstrate that the age-related changes in DNA methylation play a mechanistic role in aging (15). Importantly, aging is associated with increased incidence, delayed recovery, and worse outcomes of sepsis (34, 35). Thus, we hypothesized that age-related changes in DNA methylation may play a role in the susceptibility and prognosis of sepsis. Indeed, 241 CpG sites that possessed significant differences in DNA methylation between sepsis patients and controls in a direction consistent with the age-related change, meaning that these 241 age-related CpG sites may be the potential mechanisms underlying the increased risk of sepsis in older adults. Interestingly, genes distributed by these CpG sites were mainly involved in TGF-β signaling pathway, which is essential for LPS-induced sepsis (26). Moreover, inhibiting TGF-β pathway can ameliorate sepsis-induced organ dysfunction and increase survival time of septic mice (36, 37). In addition, 161 CpG sites that possessed significant differences in DNA methylation between survivors and non-survivors in a direction consistent with the age-related change, supporting that 161 age-related CpG sites may play a crucial role in the worse outcomes of elderly septic patients. Importantly, the top enriched pathway among these CpG sites distributed genes was mTOR signaling pathway, which plays an important role in immunosuppression following sepsis (30). Similarly, several studies have indicated that inhibiting mTOR signaling pathway can attenuate sepsis-induced organ dysfunction in rats (38, 39). Additionally, mTOR signaling is strongly implicated in aging (31). The mTOR inhibitor rapamycin is now the only known pharmacological intervention that can extend lifespan in all tested animal models (40–43). One possible underlying mechanism is that inhibition of mTOR complex 1 (mTORC1) may stimulate autophagy, which helps clear damaged mitochondria, the accumulation of which are linked to aging and aging-related diseases such as sepsis (40). Accordingly, our findings open new perspectives for reducing the incidence and mortality of sepsis, especially in elderly patients.

Given that DNA methylation in cytokine genes changes upon aging (44–46), we analyzed inflammatory cytokine genes, TNF-α, IL-1β, IL-6, and IL-8, which includes a total of 72 CpG sties. Interestingly, 6 sites (8.3%) were correlated with age in sepsis patients, and the CpG site with the most significant correlation was in TNF-α (Supplementary Figure 3A). Moreover, this site was hypermethylated in non-survivors (Supplementary Figure 3B), which is consistent with previous studies that increased methylation in TNF gene was associated with poorer survival in pancreatic cancer (47). Thus, our findings indicated that DNA methylation changes in TNF-α gene along with aging may play a role in sepsis progression. However, methylation of TNF have been found to be negative correlated to age in healthy controls (48). We next explored the correlation between methylation of TNF and age in our control group, and a total of 25 CpG sites were included in our methylation data. All of these sites showed negative correlation along with age (minimum *p*-value was found in cg17741993, *p* = 0.09), although there was no statistical significance, which may be attributed to the small sample size.

One potential limitation of our study is the size of the discovery cohort. In order to alleviate this limitation and further verify our findings, we validated the selected CpG site with a larger sample size. Another limitation is that we were unable to test the relation of identified age-related DNA methylation changes with expression of respective nearby annotated genes. Since DNA methylation is often associated with gene expression (49), future studies are needed to explore expression of genes with age-related DNA methylation changes, and further investigated exact mechanisms of these genes in the occurrence and progression of sepsis.

In conclusion, our study revealed for the first time that age-related changes in DNA methylation may play a role in the disproportionate susceptibility and worse outcomes of sepsis in older adults, and provide a new insight into the potential link among sepsis, epigenetic changes, and aging.

## Data Availability Statement

The data presented in the study are deposited in the OMIX, China National Center for Bioinformation/Beijing Institute of Genomics, Chinese Academy of Sciences (https://ngdc.cncb.ac.cn/omix/release/OMIX904), accession number OMIX904.

## Ethics Statement

The studies involving human participants were reviewed and approved by the Research Ethics Committee of the First Affiliated Hospital, College of Medicine, Zhejiang University. Written informed consent to participate in this study was provided by the participants or their legal guardian/next of kin.

## Author Contributions

JC, HJ, and XL designed the study. LS, TZ, and QS provided the samples. WZ, YC, and CZ carried out experiments. CW, YW, and FN performed the statistical analysis. XL and LS wrote the manuscript. All authors contributed to the manuscript and approved the submitted version.

## Funding

This work was supported by Natural Science Foundation of China (81900611, LQ19H050008, and 2019313917).

## Conflict of Interest

The authors declare that the research was conducted in the absence of any commercial or financial relationships that could be construed as a potential conflict of interest.

## Publisher's Note

All claims expressed in this article are solely those of the authors and do not necessarily represent those of their affiliated organizations, or those of the publisher, the editors and the reviewers. Any product that may be evaluated in this article, or claim that may be made by its manufacturer, is not guaranteed or endorsed by the publisher.

## References

[1] Cecconi M Evans L Levy M Rhodes A. Sepsis and septic shock. The Lancet. (2018) 392:75–87. 10.1016/S0140-6736(18)30696-2 29937192

[2] Kaukonen KM Bailey M Suzuki S Pilcher D Bellomo R. mortality related to severe sepsis and septic shock among critically ill patients in Australia and New Zealand, 2000–2012. JAMA. (2014) 311:1308–16. 10.1001/jama.2014.2637 24638143

[3] Iwashyna TJ Cooke CR Wunsch H Kahn JM. Population burden of long-term survivorship after severe sepsis in older Americans. J Am Geriatr Soc. (2012) 60:1070–7. 10.1111/j.1532-5415.2012.03989.x 22642542 PMC3374893

[4] Rowe TA McKoy JM. Sepsis in older adults. Infect Dis Clin North Am. (2017) 31:731–42. 10.1016/j.idc.2017.07.010 29079157

[5] Nasa P Juneja D Singh O Dang R Arora V. Severe sepsis and its impact on outcome in elderly and very elderly patients admitted in intensive care unit. J Intensive Care Med. (2012) 27:179–83. 10.1177/0885066610397116 21436163

[6] Bermejo-Martin JF Martin-Fernandez M Lopez-Mestanza C Duque P Almansa R. Shared features of endothelial dysfunction between sepsis and its preceding risk factors (aging and chronic disease). J Clin Med. (2018) 7:11. 10.3390/jcm7110400 30380785 PMC6262336

[7] Robertson KD. DNA methylation and human disease. Nat Rev Genet. (2005) 6:597–610. 10.1038/nrg1655 16136652

[8] Binnie A Tsang JLY Hu P Carrasqueiro G Castelo-Branco P Dos Santos CC. Epigenetics of sepsis. Crit Care Med. (2020) 48:745–56. 10.1097/CCM.0000000000004247 32167492

[9] Falcao-Holanda RB Brunialti MKC Jasiulionis MG Salomao R. Epigenetic regulation in sepsis, role in pathophysiology and therapeutic perspective. Front Med (Lausanne). (2021) 8:685333. 10.3389/fmed.2021.685333 34322502 PMC8312749

[10] Binnie A Walsh CJ Hu P Dwivedi DJ Fox-Robichaud A Liaw PC. Epigenetic profiling in severe sepsis: a pilot study of DNA methylation profiles in critical illness. Crit Care Med. (2020) 48:142–50. 10.1097/CCM.0000000000004097 31939781

[11] Lorente-Pozo S Navarrete P Garzon MJ Lara-Canton I Beltran-Garcia J Osca-Verdegal R. DNA methylation analysis to unravel altered genetic pathways underlying early onset and late onset neonatal sepsis. a pilot study. Front Immunol. (2021) 12:622599. 10.3389/fimmu.2021.622599 33659006 PMC7917190

[12] Lorente-Sorolla C Garcia-Gomez A Catala-Moll F Toledano V Ciudad L Avendano-Ortiz J. Inflammatory cytokines and organ dysfunction associate with the aberrant DNA methylome of monocytes in sepsis. Genome Med. (2019) 11:66. 10.1186/s13073-019-0674-2 31665078 PMC6820973

[13] Cao L Zhu T Lang X Jia S Yang Y Zhu C. Inhibiting DNA methylation improves survival in severe sepsis by regulating nf-kappab pathway. Front Immunol. (2020) 11:1360. 10.3389/fimmu.2020.01360 32714333 PMC7343767

[14] Field AE Robertson NA Wang T Havas A Ideker T Adams PD. DNA methylation clocks in aging: categories, causes, and consequences. Mol Cell. (2018) 71:882–95. 10.1016/j.molcel.2018.08.008 30241605 PMC6520108

[15] Unnikrishnan A Freeman WM Jackson J Wren JD Porter H Richardson A. The role of DNA methylation in epigenetics of aging. Pharmacol Ther. (2019) 195:172–85. 10.1016/j.pharmthera.2018.11.001 30419258 PMC6397707

[16] Bacos K Gillberg L Volkov P Olsson AH Hansen T Pedersen O. Blood-based biomarkers of age-associated epigenetic changes in human islets associate with insulin secretion and diabetes. Nat Commun. (2016) 7:11089. 10.1038/ncomms11089 27029739 PMC4821875

[17] Heylen L Thienpont B Busschaert P Sprangers B Kuypers D Moisse M. Age-related changes in DNA methylation affect renal histology and post-transplant fibrosis. Kidney Int. (2019) 96:1195–204. 10.1016/j.kint.2019.06.018 31530476

[18] Xu Z Taylor JA. Genome-wide age-related DNA methylation changes in blood and other tissues relate to histone modification, expression and cancer. Carcinogenesis. (2014) 35:356–64. 10.1093/carcin/bgt391 24287154 PMC3908753

[19] Kwabi-Addo B Chung W Shen L Ittmann M Wheeler T Jelinek J. Age-related DNA methylation changes in normal human prostate tissues. Clin Cancer Res. (2007) 13:3796–802. 10.1158/1078-0432.CCR-07-0085 17606710

[20] Johnson KC Koestler DC Cheng C Christensen BC. Age-related DNA methylation in normal breast tissue and its relationship with invasive breast tumor methylation. Epigenetics. (2014) 9:268–75. 10.4161/epi.27015 24196486 PMC3962537

[21] Singer M Deutschman CS Seymour CW Shankar-Hari M Annane D Bauer M. The third international consensus definitions for sepsis and septic shock (Sepsis-3). JAMA. (2016) 315:801–10. 10.1001/jama.2016.0287 26903338 PMC4968574

[22] Phipson B Maksimovic J Oshlack A. missMethyl: an R package for analyzing data from illumina's humanmethylation450 platform. Bioinformatics. (2016) 32:286–8. 10.1093/bioinformatics/btv560 26424855

[23] van der Poll T van de Veerdonk FL Scicluna BP Netea MG. The immunopathology of sepsis and potential therapeutic targets. Nat Rev Immunol. (2017) 17:407–20. 10.1038/nri.2017.36 28436424

[24] Yan X Tu H Liu Y Chen T Cao J. Interleukin-17D aggravates sepsis by inhibiting macrophage phagocytosis. Crit Care Med. (2020) 48:e58–E65. 10.1097/CCM.0000000000004070 31634237

[25] Sun J Zhang J Tian J Virzi GM Digvijay K Cueto L. Mitochondria in sepsis-induced AKI. J Am Soc Nephrol. (2019) 30:1151–61. 10.1681/ASN.2018111126 31076465 PMC6622414

[26] Zhang C Li J Qiu X Chen Y Zhang X. SUMO protease SENP1 acts as a CeRNA for TGFBR2 and Thus Activates TGFBR2/Smad signaling responsible for LPS-induced sepsis. Biomed Pharmacother. (2019) 112:108620. 10.1016/j.biopha.2019.108620 30797150

[27] Neviere R Delguste F Durand A Inamo J Boulanger E Preau S. Abnormal Mitochondrial CAMP/PKA signaling is involved in sepsis-induced mitochondrial and myocardial dysfunction. Int J Mol Sci. (2016) 17:12. 10.3390/ijms17122075 27973394 PMC5187875

[28] Jin P Deng S Tian M Lenahan C Wei P Wang Y. INT-777 prevents cognitive impairment by activating takeda G protein-coupled receptor 5 (TGR5) and attenuating neuroinflammation *via* CAMP/ PKA/ CREB signaling axis in a rat model of sepsis. Exp Neurol. (2021) 335:113504. 10.1016/j.expneurol.2020.113504 33058889

[29] Ginde AA Moss M Shapiro NI Schwartz RS. Impact of older age and nursing home residence on clinical outcomes of US emergency department visits for severe sepsis. J Crit Care. (2013) 28:606–11. 10.1016/j.jcrc.2013.03.018 23683561 PMC3770757

[30] Kim EY Ner-Gaon H Varon J Cullen AM Guo J Choi J. Post-sepsis immunosuppression depends on NKT cell regulation of MTOR/IFN-Gamma in NK Cells. J Clin Invest. (2020) 130:3238–52. 10.1172/JCI128075 32154791 PMC7260006

[31] Liu GY Sabatini DM. MTOR at the nexus of nutrition, growth, aging and disease. Nat Rev Mol Cell Biol. (2020) 21:183–203. 10.1038/s41580-019-0199-y 31937935 PMC7102936

[32] Gilchrist JJ Mentzer AJ Rautanen A Pirinen M Mwarumba S Njuguna P. Genetic Variation in VAC14 is associated with bacteremia secondary to diverse pathogens in African children. Proc Natl Acad Sci U S A. (2018) 115:E3601–E3. 10.1073/pnas.1802071115 29588414 PMC5910872

[33] Unnikrishnan A Hadad N Masser DR Jackson J Freeman WM Richardson A. Revisiting the genomic hypomethylation hypothesis of aging. Ann N Y Acad Sci. (2018) 1418:69–79. 10.1111/nyas.13533 29363785 PMC5934293

[34] Barter J Kumar A Stortz JA Hollen M Nacionales D Efron PA. Age and sex influence the hippocampal response and recovery following sepsis. Mol Neurobiol. (2019) 56:8557–72. 10.1007/s12035-019-01681-y 31278440 PMC6834928

[35] Banerjee D Opal SM. Age, exercise, and the outcome of sepsis. Crit Care. (2017) 21:286. 10.1186/s13054-017-1840-9 29169402 PMC5701382

[36] Cao X Zhang C Zhang X Chen Y Zhang H. MiR-145 negatively regulates TGFBR2 signaling responsible for sepsis-induced acute lung injury. Biomed Pharmacother. (2019) 111:852–8. 10.1016/j.biopha.2018.12.138 30841464

[37] Zhou YX Han WW Song DD Li ZP Ding HJ Zhou T. Effect of miR-10a on sepsis-induced liver injury in rats through TGF-Beta 1/Smad signaling pathway. Eur Rev Med Pharmaco. (2020) 24:862–9. 32016992 10.26355/eurrev_202001_20070

[38] Zhuang X Yu Y Jiang Y Zhao S Wang Y Su L. Molecular hydrogen attenuates sepsis-induced neuroinflammation through regulation of microglia polarization through an MTOR-autophagy-dependent pathway. Int Immunopharmacol. (2020) 81:106287. 10.1016/j.intimp.2020.106287 32058932

[39] Shi X Liu Y Zhang D Xiao D. Valproic acid attenuates sepsis-induced myocardial dysfunction in rats by accelerating autophagy through the PTEN/AKT/MTOR pathway. Life Sci. (2019) 232:116613. 10.1016/j.lfs.2019.116613 31265853

[40] Saxton RA Sabatini DM. mTOR signaling in growth, metabolism, and disease. Cell. (2017) 168:960–76. 10.1016/j.cell.2017.02.004 28283069 PMC5394987

[41] Powers RW 3rd Kaeberlein M Caldwell SD Kennedy BK Fields S. Extension of chronological life span in yeast by decreased TOR pathway signaling. Genes Dev. (2006) 20:174–84. 10.1101/gad.1381406 16418483 PMC1356109

[42] Bjedov I Toivonen JM Kerr F Slack C Jacobson J Foley A. Mechanisms of life span extension by rapamycin in the fruit fly drosophila melanogaster. Cell Metab. (2010) 11:35–46. 10.1016/j.cmet.2009.11.010 20074526 PMC2824086

[43] Harrison DE Strong R Sharp ZD Nelson JF Astle CM Flurkey K. Rapamycin fed late in life extends lifespan in genetically heterogeneous mice. Nature. (2009) 460:392–5. 10.1038/nature08221 19587680 PMC2786175

[44] Matt SM Lawson MA Johnson RW. Aging and peripheral lipopolysaccharide can modulate epigenetic regulators and decrease IL-1beta Promoter DNA methylation in microglia. Neurobiol Aging. (2016) 47:1–9. 10.1016/j.neurobiolaging.2016.07.006 27500965 PMC5075520

[45] Yamanashi T Saito T Yu T Alario A Comp K Crutchley KJ. DNA methylation in the TNF-alpha gene decreases along with aging among delirium inpatients. Neurobiol Aging. (2021) 105:310–7. 10.1016/j.neurobiolaging.2021.05.005 34192631

[46] Shinozaki G Braun PR Hing BWQ Ratanatharathorn A Klisares MJ Duncan GN. Epigenetics of delirium and aging: potential role of DNA methylation change on cytokine genes in glia and blood along with aging. Front Aging Neurosci. (2018) 10:311. 10.3389/fnagi.2018.00311 30405391 PMC6206747

[47] Huang BZ Binder AM Sugar CA Chao CR Setiawan VW Zhang ZF. Methylation of immune-regulatory cytokine genes and pancreatic cancer outcomes. Epigenomics. (2020) 12:1273–85. 10.2217/epi-2019-0335 32867538 PMC7546161

[48] Gowers IR Walters K Kiss-Toth E Read RC Duff GW Wilson AG. Age-related loss of CpG methylation in the tumor necrosis factor promoter. Cytokine. (2011) 56:792–7. 10.1016/j.cyto.2011.09.009 22004920

[49] Jones PA. Functions of DNA methylation: islands, start sites, gene bodies and beyond. Nat Rev Genet. (2012) 13:484–92. 10.1038/nrg3230 22641018

