# Supplementary material for: Role of Age-Related Changes in DNA Methylation in the Disproportionate Susceptibility and Worse Outcomes of Sepsis in Older Adults
Source: Front Med (Lausanne). 2022 Feb 15;9:822847. doi: 10.3389/fmed.2022.822847 (PMC8886726; doi:10.3389/fmed.2022.822847)
Supplement: Supplementary file 9 [file Data_Sheet_1.PDF]

Supplementary Material

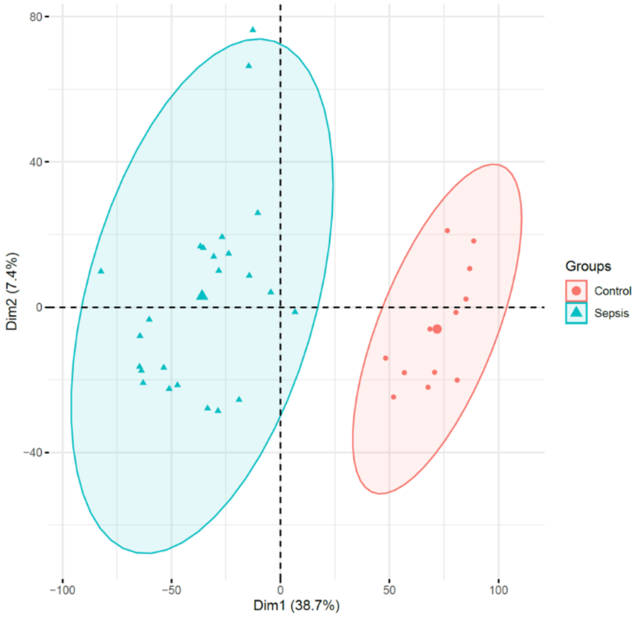

Supplementary Figure 1. Principal component analysis (PCA) of methylation data of methylation variable positions (MVPs) in sepsis and control group (in blue and red respectively).

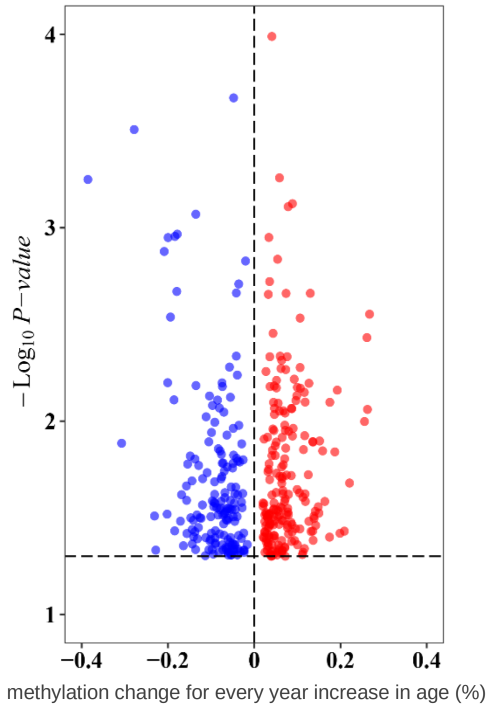

Supplementary Figure 2. Volcano plot of age-related CpG sites (n=366) in controls.

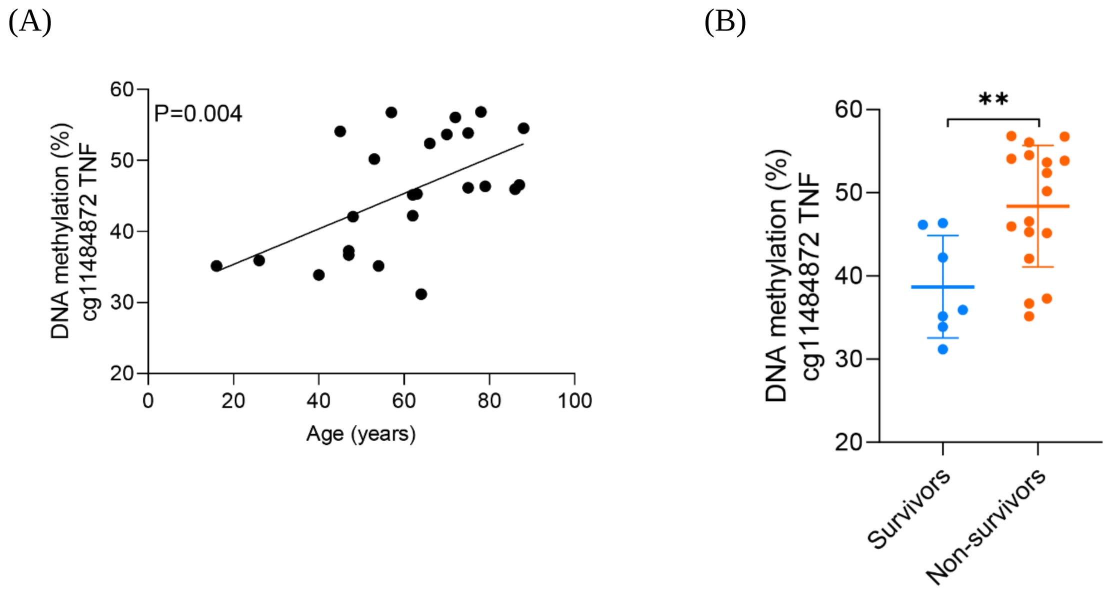

Supplementary Figure 3. DNA methylation of the CpG site (cg11484872) in TNF. (A) Correlation between age and methylation of the CpG site (cg11484872) in TNF as analysed by linear regression analysis (n=24). (B) 24 sepsis patients were divided into two groups (7 survivors and 17 non-survivors) according to their hospital outcomes. Data shown are mean ± SD. **P < 0.01. Comparisons between two groups were done by student's t-test.
